# ﻿Comparative cytogenetics of the *Physalaemus gracilis* group (Anura, Leptodactylidae) with characterization of the karyotype of *Physalaemus evangelistai* Bokermann, 1967

**DOI:** 10.3897/compcytogen.19.171637

**Published:** 2025-11-07

**Authors:** Pedro Henrique Pacheco Mosquini, Lucas Henrique Bonfim Souza, Juan Martín Ferro, Luciana Bolsoni Lourenço

**Affiliations:** 1 Laboratório de Estudos Cromossômicos, Departamento de Biologia Estrutural e Funcional, Instituto de Biologia, Universidade Estadual de Campinas (LabEsC – UNICAMP), 13083-863 Campinas, São Paulo, Brasil Universidade Estadual de Campinas (LabEsC – UNICAMP) Campinas Brazil; 2 Laboratorio de Genética Evolutiva, Instituto de Biología Subtropical (CONICET-UNaM), Facultad de Ciencias Exactas Químicas y Naturales, Universidad Nacional de Misiones, Félix de Azara 1552, CPA N3300LQF, Posadas, Misiones, Argentina Universidad Nacional de Misiones Posadas Argentina

**Keywords:** C-band, karyotype, Leptodactylidae, NOR, PcP190 satellite DNA

## Abstract

The anuran species group *Physalaemus gracilis* comprises six species, and variation in the location of nucleolus organizer regions (NORs) was observed across the four species that have been karyotyped to date. The NORs are located interstitially on chromosome 8 of *P. carrizorum* Cardozo et Pereyra, 2018, and *P. lisei* Braun et Braun, 1977, terminally on chromosome 8 of *P. gracilis* (Boulenger, 1883), and terminally on chromosome 10 of *P. barrioi* Bokermann, 1967. To contribute to the comparative analysis of this group, including the assessment of the hypothesis of homology among these NOR-bearing chromosomes, we described the karyotype of *P. evangelistai* Bokermann, 1967, and expanded the cytogenetic analyses of *P. carrizorum*, *P. lisei*, and *P. barrioi*. We used classical cytogenetic techniques and mapped, by fluorescent in situ hybridization (FISH), two repetitive sequences: the PcP190 satellite DNA and the U2 snRNA gene. *Physalaemus evangelistai* exhibited a 2n = 22 karyotype, with meta- and submetacentric chromosomes, which corresponds to the typical karyotypic configuration of the genus. We found an interstitial heterochromatin DAPI-positive band on the short arm of the NOR-bearing chromosomes 8 of *P. evangelistai* and *P. carrizorum* from Palmas-PR, and chromosome 10 of *P. barrioi*, which corroborates the hypothesis that these chromosomes are homologous. In *P. evangelistai*, an additional NOR was observed on chromosome 9 of females. Moreover, the karyotype of *P. carrizorum* from Palmas-PR differed from that previously described for *P. carrizorum* from Misiones, particularly in the number of PcP190 clusters and intrachromosomal position of the NOR on chromosome 8. Specimens from Palmas-PR showed a terminal NOR on chromosome 8 and PcP190 clusters on chromosomes 1 and 3, whereas those from Misiones had an interstitial/pericentromeric NOR on chromosome 8 and a single PcP190 cluster on chromosome 3. Further analyses are still needed to assess whether these cytogenetic differences represent interspecific variation.

## ﻿Introduction

Karyotype comparisons can be highly informative for distinguishing certain clades, as they provide characters related to diploid number, chromosomal morphology and size, the distribution of repetitive elements, and other traits that may be specific to distinct lineages (Dobigny et al. 2004; Guerra 2008; Mezzasalma et al. 2023). The Neotropical genus *Physalaemus* Fitzinger, 1826 represents an interesting clade in terms of chromosome evolution, and karyotype data have proven informative for comparing phylogenetically related groups within this genus (see Tomatis et al. 2009; Vittorazzi et al. 2014b; Lourenço et al. 2015; Ferro et al. 2022; Souza et al. 2025 and references therein). In addition, this genus is characterized by a high frequency of chromosomal polymorphisms and a large number of cryptic species (e.g., Barrio 1965; Cassini et al. 2010; Nascimento et al. 2019), which often hinder species identification based solely on morphological characteristics.

The anuran genus *Physalaemus*, which belongs to the family Leptodactylidae and comprises 50 species, is distributed from southern Venezuela and the Guianas to eastern Bolivia, Paraguay, southeastern Colombia, Uruguay, and a large portion of Brazil (Frost 2025). Phylogenetic inferences divide this genus into two major clades: the *Physalaemus signifer* clade and the *Physalaemus cuvieri* clade (Lourenço et al. 2015). The latter clade encompasses five species groups—*P. gracilis*, *P. biligonigerus*, *P. cuvieri*, *P. henselii*, and *P. olfersii*—as well as the species *Physalaemus cicada* Bokermann, 1966, which has not been allocated to any group (Lourenço et al. 2015).

Species of *Physalaemus* exhibit karyotypes with 2n = 22 and fundamental numbers (FN) of 42 or 44 (see discussion in Tomatis et al. 2009; Lourenço et al. 2015). The karyotypes of all karyotyped species of the *P. signifer* Clade have FN = 42 and are characterized by the presence of a telocentric chromosome pair 11, which was hypothesized to be a synapomorphy of this clade (Lourenço et al. 2015). Another notable cytogenetic feature in this genus is a heterochromatic band on the short arm of chromosome 5, which may represent a synapomorphy of the *Physalaemus cuvieri* group (Vittorazzi et al. 2014b; Lourenço et al. 2015).

The *Physalaemus gracilis* group is composed of six species: *P. lisei* Braun et Braun, 1977, *P. gracilis* (Boulenger, 1883), *P. evangelistai* Bokermann, 1967, *P. carrizorum* Cardozo et Pereyra, 2018, *P. jordanensis* Bokermann, 1967, and *P. barrioi* Bokermann, 1967 (Lourenço et al. 2015; Cardozo and Pereyra 2018). Four out of these six species have had their karyotypes previously described (Brum-Zorrilla and Saez 1968; De Lucca et al. 1974; Provete et al. 2012; Ferro et al. 2022), while the karyotypes of *P. evangelistai* and *P. jordanensis* remain unknown. Across the karyotyped species, variation in the position of the nucleolus organizer region (NOR) is observed: it is interstitial on chromosome 8 of *P. carrizorum* and *P. lisei*, terminal on chromosome 8 of *P. gracilis*, and terminal on chromosome 10 of *P. barrioi* (Brum-Zorrilla and Saez 1968; De Lucca et al. 1974; Provete et al. 2012; Ferro et al. 2022). Given that these NOR-bearing chromosomes are also similar in morphology and size, Ferro et al. (2022) proposed that they are homologous. However, the absence of further cytogenetic markers still prevents a comprehensive evaluation of this hypothesis.

Other interesting chromosomal markers observed in the *P. gracilis* group are sites enriched with the PcP190 satellite DNA (satDNA) (Vittorazzi et al. 2011), which is a repetitive DNA sequence widely found in anurans, reported in seven families of Hyloidea to date (Vittorazzi et al. 2011, 2014a; Gatto et al. 2016, 2018; Targueta et al. 2018; Gatto et al. 2019). In the analyzed species of the *P. gracilis* group, Ferro et al. (2022) identified a small number of chromosomal clusters of PcP190 per karyotype, distributed in apparently species-specific patterns. While in *P. lisei* a cluster of PcP190 was mapped to the (peri)centromeric region of chromosome 7, in *P. carrizorum* it was found on chromosome 3, and in *P. gracilis*, on chromosomes 2 and 9 (Ferro et al. 2022).

To enhance our understanding of chromosomal homologies and evolutionary relationships within the *P. gracilis* group, we described the karyotype of *P. evangelistai*, presented new data for *P. carrizorum* from a newly sampled locality, and conducted a comparative analysis of repetitive DNA clusters among the species with available chromosomal data in this species group.

## ﻿Material and methods

### ﻿Species, chromosome preparations, and conventional techniques

We analyzed samples of *P. barrioi*, *P. carrizorum*, *P. evangelistai*, *P. gracilis*, and *P. lisei* (Table 1). *Physalaemus evangelistai* specimens and *P. carrizorum* samples from Palmas, state of Paraná, were collected under the authorization of the Chico Mendes Institute for Biodiversity Conservation/Biodiversity and Information System (ICMBio/SISBIO - permit number 32483). The sampling sites and voucher numbers in scientific collections are provided for all analyzed specimens in Table 1.

**Table 1.** Sampling sites and voucher numbers in zoological and cytogenetic collections of the analyzed specimens. MG – Minas Gerais state, PR – Paraná state, RS – Rio Grande do Sul state, SP – São Paulo state, CFBH – Coleção de Anfíbios Célio Fernando Baptista Haddad, Universidade Estadual Paulista, Rio Claro, SP, Brasil. LGE – Laboratorio de Genética Evolutiva, Instituto de Biología Subtropical (CONICET-UNaM), Posadas, Misiones, Argentina. ZUEC – Museu de Diversidade Biológica, Universidade Estadual de Campinas, Campinas, SP, Brasil. SMRP – Collection of tissue and chromosome preparation Shirlei Maria Recco Pimentel, LabEsC-UNICAMP, Campinas, SP, Brasil. ^1^Specimen collected by Provete et al. (2012). ^2^Material analyzed by Ferro et al. (2022) and reanalyzed in this work. ^3^The sample LGE 24608 corresponds to the same specimen labeled as LGE 24602 in Ferro et al. (2022), where the number was a typographical error.

Species	Locality	Voucher number in zoological collection	Chromosome preparation accession number
*Physalaemus barrioi*	São José do Barreiro, SP, Brasil (22°43'33"S, 44°37'16"W) ^1^	ZUEC 18146 ♂	SMRP 303.1
	Piraquara, PR, Brasil (25°29'47"S, 48°58'54"W)	LGE 15329	LGE 15329
*Physalaemus carrizorum*	Palmas, PR, Brasil (26°33'21"S, 51°39'41"W)	ZUEC 24848 ♂, ZUEC 24853 ♂	SMRP 551.1, SMRP 551.2
	Arroyo de los Muertos, Misiones, Argentina (27°22'12"S, 54°24'30"W) ^2^	LGE 24608 ♂ ^3^	LGE 24608 ^3^
*Physalaemus evangelistai*	Salinas, MG, Brasil (16°5'55"S, 42°12'22"W)	CFBH 45513 ♂, CFBH 45514 ♀, CFBH 45515 ♂, CFBH 45516 ♂, CFBH 45517 ♂, CFBH 45518 ♀	SMRP 554.1, SMRP 554.2, SMRP 554.3, SMRP 554.4, SMRP 554.5, SMRP 554.6
*Physalaemus gracilis*	Gravataí, RS, Brasil (29°48'9"S, 50°55'50"W) ^2^	LGE 28043 ♂	SMRP 37.19
*Physalaemus lisei*	Gramado, RS, Brasil (29°21'8"S, 50°53'7"W)	ZUEC 24003 ♂	SMRP 37.15

Chromosome preparations were obtained from cell suspensions deposited in the scientific collection “Shirlei Maria Recco Pimentel” (SMRP), housed at LabEsC-UNICAMP. These suspensions were previously prepared from intestinal and testicular tissues, following, respectively, the protocols of King and Rofe (1976), with modifications outlined by Gatto et al. (2018), and Schmid (1978), replacing the hypotonic KCl solution with cold distilled water. All experiments were conducted in accordance with relevant guidelines and approved by the Committee for Ethics in Animal Use of the University of Campinas (CEUA/UNICAMP).

Mitotic metaphases were stained with 10% Giemsa, C-banded following Sumner (1972), with modifications described in Siqueira et al. (2008), and silver-impregnated using the Ag-NOR method following Howell and Black (1980). Some C-banded metaphases were sequentially stained with 4’-6 diamidine-2-phenylindole (DAPI, 0.5 µg/mL) and Chromomycin A_3_ (CMA_3_, 0.5 mg/mL) after removal of Giemsa-staining in 50% acetic acid for five minutes. Mitotic metaphases were observed using an Olympus BX-60 microscope (Olympus, Tokyo, Japan). Images were captured with a Quick Start FL-20 camera, using Mosaic 2.4 (Tucsen Photonics, Fuzhou, China). Brightness and contrast adjustments were made using Adobe Photoshop 2021 (Adobe Systems, San Jose, CA, USA).

### ﻿Isolation and analysis of nucleotide sequences of PcP190 satellite DNA

Genomic DNA samples were obtained from liver tissue of one specimen of *P. evangelistai* (CFBH 45518) and one specimen of *P. lisei* (ZUEC 24003). DNA extraction was performed following the TNES method as employed by Medeiros et al. (2013), or alternatively using the QIAamp Fast DNA Tissue Kit (QIAGEN, Hilden, Germany), following the manufacturer’s instructions. The PcP190 satDNA was isolated by PCR using the specific set of primers P190F and P190R (Vittorazzi et al. 2011). The PCR products were purified using the Wizard SV Gel and PCR Clean-Up System (Promega Corporation, Madison, WI, USA), following the manufacturer’s instructions. Samples of the purified products were sequenced by the sequencing facility of the Human Genome and Stem Cell Research Center/IB-USP. The obtained sequences were edited using the BioEdit Sequence Alignment Editor v.7.7.1 (Hall 1999) and compared with sequences available in the NCBI database using BLASTn. After BLASTn indicated similarity between the isolated sequences and those of PcP190 type 1a (see Gatto et al. 2016 for details), we aligned the obtained sequences with all complete PcP190 1a monomers, available in the NCBI database. We excluded monomers derived from *P. albonotatus* (Steindachner, 1864), which has a 7 bp deletion. The average p-distance value was calculated using the MEGA v.11.0.13 software (Tamura et al. 2021), treating alignment gaps and missing data as pairwise deletions. Alignment figures were generated using Geneious v.7.1.3 (https://www.geneious.com).

### ﻿Fluorescent in situ Hybridization (FISH) of PcP190 Satellite DNA and U2 snRNA gene

PcP190 satDNA and U2 snRNA gene sequences were obtained from cloned fragments available in the SMRP collection, previously isolated from *Physalaemus cuvieri* Fitzinger, 1826 (KM361675.1, Vittorazzi et al. [2014a]) and *P. ephippifer* (Steindachner, 1864) (PQ200500, Souza et al. [2025]), respectively. The PcP190 satDNA was labeled using a PCR Dig Probe Synthesis Kit (Roche) with the universal primers SP6 and T7. The U2 snRNA gene probe was obtained using the primers U2-F and U2-R, designed by Souza et al. (2025). The resulting probes were hybridized with chromosome preparations following the protocol by Viegas-Péquignot (1992). The probes were detected using an anti-digoxigenin-rhodamine antibody (0.06 μg/mL), the chromosomes were counterstained with DAPI (0.5 μg/mL), and the preparations were mounted in Vectashield Antifade Mounting Medium (Vector Laboratories, Burlingame, CA, USA). Microscope analyses and image acquisition were performed as mentioned above.

## ﻿Results

### ﻿The karyotype of *Physalaemus evangelistai*

**Figure 1.**
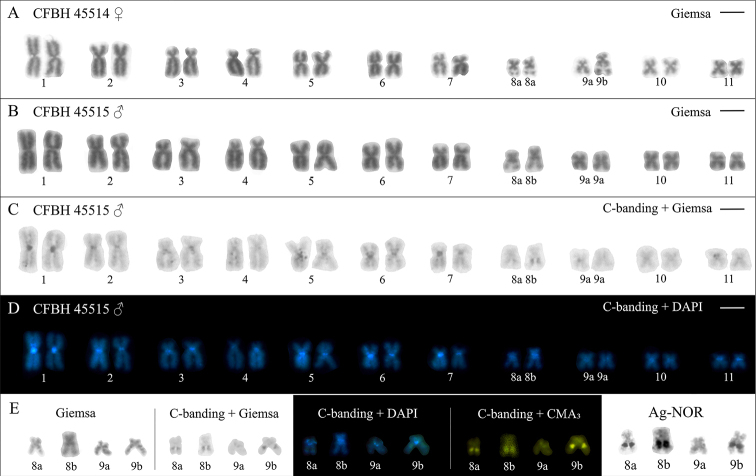
Chromosomes of *Physalaemus evangelistai***A** female karyotype stained with Giemsa **B–D** male karyotype sequentially subjected to Giemsa-staining (**B**), conventional C-banding (**C**), and DAPI-staining (**D**) **E** chromosomes 8 and 9 subjected to Giemsa-staining, conventional C-banding, C-banding with DAPI- and CMA₃-stainings, and silver-impregnation using the Ag-NOR method. Scale bars: 5 µm.

The *P. evangelistai* karyotype had a diploid number of 22 and a fundamental number of 44 (Fig. 1). Chromosome pairs 1, 5, 6, and 10 were metacentric, while pairs 2–4, 7–9, and 11 were submetacentric (Fig. 1A–D). Small heterochromatin blocks were revealed by C-banding in the centromeric region of all chromosomes (Fig. 1C). All these bands were also evidenced by DAPI in metaphases previously subjected to C-banding (Fig. 1D). An interstitial C-band was found on the long arm of chromosome 8, which was DAPI-negative, CMA_3_-positive, and colocalized with the NOR (Figs 1D, E, 3B). In some metaphases, a small C-band was noted on the short arm of chromosome 8, particularly after DAPI staining (Figs 1E, 3A).

In all analyzed specimens, chromosome 8 exhibit an interstitial NOR on the long arm (Figs 1E, 3B), coinciding with a secondary constriction observed in Giemsa-stained metaphases (Figs 1E, 3B). However, variation in NOR size allowed the identification of two morphotypes: 8a, bearing a small NOR, and 8b, carrying a large NOR (Fig. 1E). Three males (CFBH 45513, CFBH 45515, and CFBH 4516) were heterozygous with respect to this condition (8a8b), while one male (CFBH 45517) was homozygous for the smaller NOR (8a8a). Both females (CFBH 45514 and CFBH 45518) were homozygous for the smaller NOR (8a8a).

In addition to chromosome pair 8, one homologue of chromosome pair 9 also carried a NOR in both analyzed females (Fig. 1E), coinciding with a secondary constriction in Giemsa-stained metaphases and being DAPI-negative and CMA_3_-positive (Fig. 1E). The NOR-bearing chromosome 9 (morph 9b) was larger than its counterpart (morph 9a), which lacks a NOR. All four analyzed males had no NOR on this chromosome pair.

**Figure 2.**
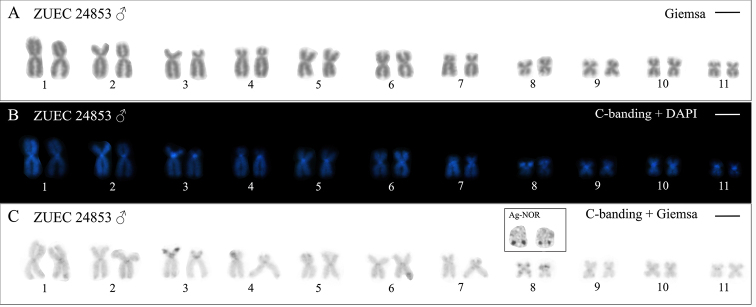
Karyotype of *Physalaemus carrizorum* from Palmas-PR **A–C** Metaphase of male ZUEC 24853 after Giemsa-staining (**A**), C-banding with DAPI-staining (**B**), and C-banding with Giemsa-staining (**C**). In the inset in C, NOR-bearing chromosomes after the Ag-NOR method. Scale bars: 5 µm.

**Figure 3.**
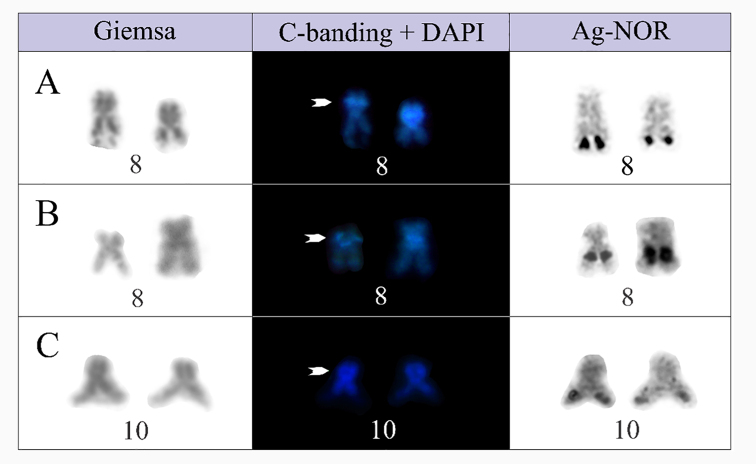
NOR-bearing chromosomes of *Physalaemus carrizorum* (**A**), *P. evangelistai* (**B**), and *P. barrioi* (**C**), sequentially subjected to Giemsa-staining, C-banding and DAPI-staining, and the Ag-NOR method. Arrowheads indicate an interstitial DAPI-positive C-band present in all species. Note that, although less evident, it is also present in the NOR-bearing chromosome of *P. barrioi*.

*Physalaemus carrizorum* from Palmas-PR presented a karyotype with FN = 44 and 2n = 22 (Fig. 2A–C). DAPI-positive C-bands were detected in all centromeric regions (Fig. 2B, C). Heterochromatic bands were also present pericentromerically on the short arm of chromosome 3 and on the long arm of chromosome 7, interstitially on the short arm of chromosome 8, and terminally on the short arm of chromosome 3 (Fig. 2B, C). This terminal C-band was larger in one homologue of chromosome pair 3 and more noticeable in C-banded metaphases stained with Giemsa (Fig. 2C). Chromosome 8 had a terminal NOR on the long arm (inset in Figs 2C, 3A), coinciding with a secondary constriction observed in Giemsa-stained metaphases (Figs 2A, 3A). This chromosome also presented a small interstitial C-band on the long arm adjacent to the NOR, barely seen in some metaphases (Fig. 2C).

Additionally, the reanalysis of *P. barrioi* specimens allowed us to identify a faint DAPI-stained C-band on the short arm of the NOR-bearing chromosome 10, in a position similar to that observed in the NOR-bearing chromosomes 8 of *P. evangelistai* and *P. carrizorum* (Fig. 3).

### ﻿Chromosomal mapping of PcP190 satDNA and the U2 snRNA gene in the *P. gracilis* group

The PcP190 satDNA nucleotide sequences isolated from specimens of *P. evangelistai* and *P. lisei* (GenBank accession numbers PV583974 and PV983975, respectively) were 87.5% similar to each other. Based on a comparison with PcP190 sequences available in GenBank, both sequences were assigned to the type 1a of PcP190 satDNA, as they were highly similar to previously described sequences of this type (Suppl. material 1). The mean p-distances between the PcP190 sequences of *P. evangelistai* and *P. lisei* and the previously described PcP190 1a sequences, excluding primer regions, were 0.0781 and 0.126, respectively (Suppl. material 1).

**Figure 4.**
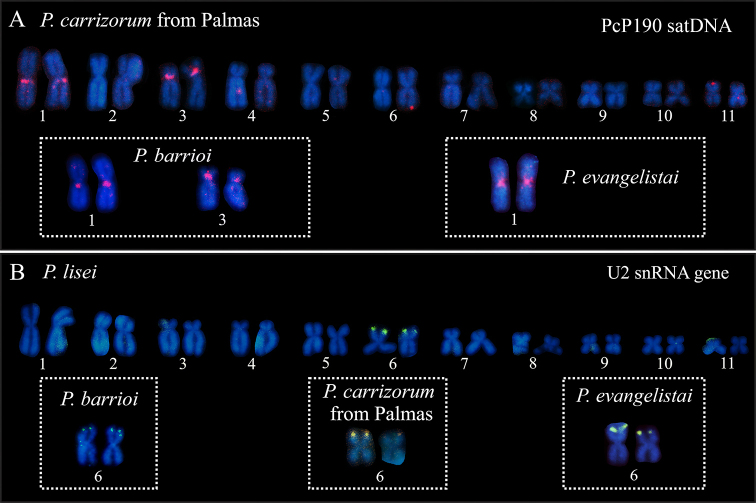
Chromosome mapping of PcP190 satDNA (in red) and U2 snRNA gene (in green) in species of the *Physalaemus gracilis* group **A** chromosomes of *P. carrizorum* from Palmas, *P. barrioi*, and *P. evangelistai* hybridized with PcP190 probe **B** mapping of the U2 snRNA gene on chromosomes of *P. lisei*, *P. barrioi*, *P. carrizorum* from Palmas, and *P. evangelistai*. Chromosomes 1 and 3 of *P. barrioi* shown in the figure were obtained from different metaphases and subsequently arranged in the figure.

Hybridization of the PcP190 satDNA probes revealed clusters of this sequence in the (peri)centromeric regions of the long arm of chromosome 1 and short arm of chromosome 3 in *P. carrizorum* from Palmas (Fig. 4A) and *P. barrioi* (inset in Fig. 4A), while in *P. evangelistai* only the short arm of chromosome 1 exhibited cluster of this sequence (inset in Fig. 4A). Chromosome mapping of the U2 snRNA gene revealed terminal clusters on the short arm of chromosome 6 in *P. barrioi*, *P. carrizorum*, *P. evangelistai*, and *P. lisei* (Fig. 4B).

## ﻿Discussion

The diploid number of *P. evangelistai* (2n = 22) is consistent with that reported for all other *Physalaemus* species karyotyped to date (Perkins et al. 2019), including members of the *P. gracilis* group (Provete et al. 2012; Ferro et al. 2022). Additionally, *P. evangelistai* has a FN of 44, a trait shared by all cytogenetically analyzed species within the *P. cuvieri* Clade, except *P. fernandezae* (Müller, 1926), which has a FN of 42 due to its smallest chromosome pair being telocentric (Tomatis et al. 2009; Lourenço et al. 2015).

All *P. evangelistai* specimens had a NOR-bearing chromosome pair 8, and an additional NOR was observed on one of the homologues of chromosome pair 9 in the two females analyzed. This additional NOR was not observed in any of the four males analyzed, nor in any other karyotyped species of the *P. gracilis* group (Provete et al. 2012; Ferro et al. 2022, Fig. 5), which may suggest the presence of a ZZ/ZW sex determination system in this species. NORs have previously been identified as distinguishing markers between sex chromosomes in some anuran species, including *Physalaemus ephippifer* (Nascimento et al. 2010), *Gastrotheca riobambae* (Schmid et al. 1983), *Buergeria buergeri* (Schmid et al. 1993), *Hyla femoralis* (Schmid and Steinlein 2003; Wiley 2003), and *Engystomops petersi* (Targueta et al. 2010). In the cases of *B. buergeri* (Schmid et al. 1993) and *H. femoralis* (Schmid and Steinlein 2003; Wiley 2003), a NOR was the sole cytogenetic feature differentiating the sex chromosomes, being found on chromosome Z in *B. buergeri*, colocalized with heterochromatin, and on chromosome X in *H. femoralis*. On the other hand, it is worth noting that polymorphisms related to the presence/absence of NORs are relatively common among anurans (e.g., Medeiros et al. 2003; Barth et al. 2009; Zaleśna et al. 2017, among several others). Therefore, the limited size of the *P. evangelistai* sample analyzed here, particularly with respect to females, prevents us from determining whether the observed heteromorphism is sex-linked or represents an autosomal polymorphism. Further studies involving a larger number of individuals would provide valuable insights into this issue.

**Figure 5.**
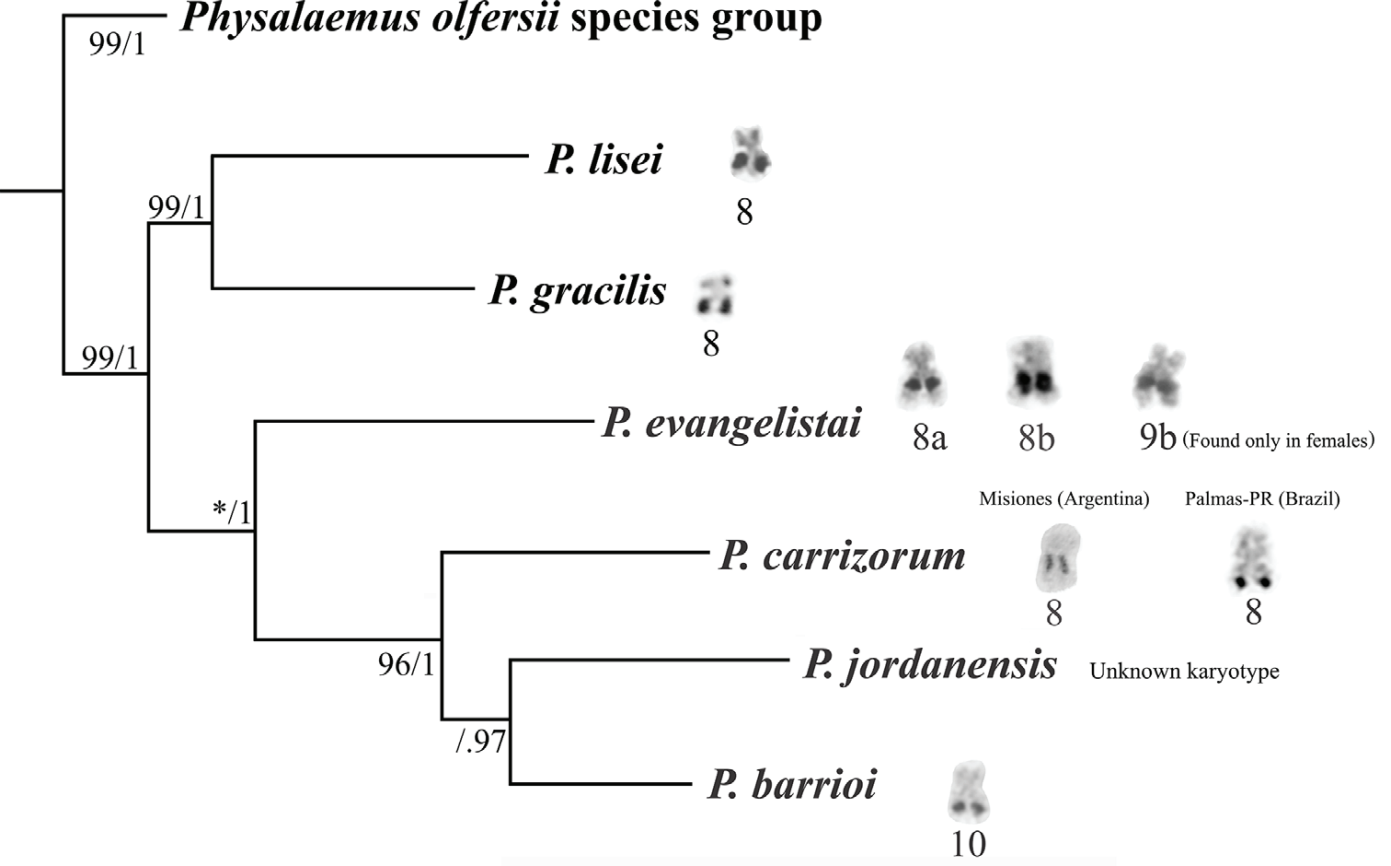
NOR-bearing chromosomes in the *P. gracilis* species group. The phylogenetic relationships are based on Lourenço et al. (2015) and Cardozo and Pereyra (2018). To date, in the *P. olfersii* group, NORs have been observed on chromosomes 3, 4, or 7, but not on chromosome 8 (Milani et al. 2010).

When comparing NOR locations across the *P. gracilis* group, some interesting patterns emerge. As in *P. evangelistai*, chromosome 8 carries a NOR in all the remaining species of the *P. gracilis* group, except *P. barrioi*, in which the NOR-bearing chromosome is chromosome 10 (Provete et al. 2012; Ferro et al. 2022, Fig. 5). In the karyotype description of *P. barrioi*, the first species of this group to have its chromosomes studied, the authors based the numerical classification of the chromosomes on their relative sizes. However, Ferro and collaborators, when describing the karyotypes of *P. gracilis*, *P. lisei*, and *P. carrizorum*, chose to designate the small metacentric chromosome bearing NOR in these species as chromosome 8, prioritizing the establishment of chromosome homology based on the presence of the NOR rather than relative size. This hypothesis, however, still demands validation, as does the possibility that the NOR-bearing chromosome of *P. barrioi* is also homologous to chromosome 8 of the other species in the group. Given that chromosomes 8–11 in each of these karyotypes are very similar in size and morphology, this analysis of homology across the species has been challenging, requiring additional chromosome markers. The presence of an interstitial C-band on the short arm of the NOR-bearing chromosomes 8 in *P. evangelistai* and *P. carrizorum* from Palmas-PR, and chromosome 10 of *P. barrioi*, as detected in this study, may support the homology of these chromosomes (see Fig. 5). Considering it is a small band, hardly revealed in some C-banded metaphases, we cannot discard the possibility that it is also present on chromosome 8 of *P. lisei* and *P. gracilis*, which comprise the sister clade of the remaining four species of the *P. gracilis* group, including *P. evangelistai*, *P. carrizorum*, and *P. barrioi* (see Fig. 5).

With respect to the intrachromosomal location, the NOR on chromosome 8 of *P. evangelistai* resembles that of *P. lisei* and *P. carrizorum* from Misiones, as it is interstitial on the long arm, although in *P. carrizorum* from Misiones the interstitial NOR extends up to the pericentromeric region. In a sharper contrast, the NOR is distal in *P. gracilis* (Ferro et al. 2022), *P. barrioi* (Provete et al. 2012), and in *P. carrizorum* from Palmas-PR. Assuming the hypothetical homology for these NOR-bearing chromosomes mentioned above, the observed variation could be attributed to intrachromosomal rearrangements, such as inversions and transposition. However, additional chromosomal markers are still required to further assess the extent of similarity among these NOR-bearing chromosomes and to accurately identify the mechanisms underlying the observed changes. Moreover, based on the phylogenetic relationships currently recognized for these species (see Fig. 5), it is not possible to confidently establish the apomorphic condition(s) of NOR position (i.e., interstitial, pericentromeric, or terminal), nor to infer the evolutionary direction of any putative chromosomal changes.

In addition to the difference in NOR-bearing chromosomes, other notable interpopulational variation was noted in *P. carrizorum*. The karyotype of specimens from Misiones also differs from that of individuals from Palmas-PR in the distribution of PcP190 satDNA chromosomal clusters. Taken together, these findings highlight the need for further analyses to properly assess whether these cytogenetic differences represent interspecific or intraspecific variation (see further discussion in the next section).

Another interesting feature in the karyotype of *P. carrizorum* from Palmas-PR is the terminal C-band on the short arm of chromosome 3, which varied notably in size, even between homologues of the same individual. Considering that the smaller band was barely detectable in our analysis, we cannot discard the possibility that it also exists in *P. carrizorum* from Misiones, although it was not observed in the sample analyzed by Ferro et al. (2022). Polymorphisms involving terminal C-bands are not uncommon and have been reported in other *Physalaemus* species, such as *P. centralis* (Vittorazzi et al. 2014b), *P. cuvieri* (Silva et al. 1999), and even *P. lisei*, a species of the *P. gracilis* group, which showed polymorphic terminal C-bands on chromosome 1 (Ferro et al. 2022).

When comparing the distribution of heterochromatin within the *P. gracilis* group, one notable feature is the reduced amount of constitutive heterochromatin present in all karyotyped species, which is mostly restricted to the centromeric regions (Provete et al. 2012; Ferro et al. 2022, present work). In addition, all the four previously karyotyped species show a remarkable C-band in the pericentromeric region of chromosome 3 (Provete et al. 2012; Ferro et al. 2022), a feature that was not observed in *P. evangelistai*. Nonetheless, the sequence composition of this heterochromatic band likely differs among the species that possess it, as suggested by the chromosome mapping of PcP190 satDNA. The pericentromeric band on chromosome 3 is enriched with this satDNA in *P. carrizorum* and *P. barrioi*, but not in *P. gracilis* and *P. lisei* (Ferro et al. 2022; present study, further discussion in the next section).

Finally, other remarkable non-centromeric C-bands detected in the karyotype of *P. evangelistai* were observed on chromosomes 8 and 9, colocalized with the NORs. The association between NOR and heterochromatin is common in anurans and have been reported for species from different families (examples in bufonid and hylid species in Schmid [1978], and in leptodactylid species in Vittorazzi et al. [2016]). Among the remaining karyotyped species of the *P. gracilis* group, only *P. gracilis* also exhibited NOR-associated heterochromatin (Ferro et al. 2022). The molecular composition of these regions, however, remain to be elucidated.

### ﻿PcP190 satellite DNA in the *Physalaemus gracilis* group

Heterochromatic blocks can serve as valuable markers for the identification of chromosome pairs and may even aid in inferring homologies between closely related species, as well as in distinguishing specific features of populations or species (e.g., Carvalho et al. 2005; Targueta et al. 2010; Guzmán-Markevich et al. 2022). The interstitial C-band on the short arm of the principal NOR-bearing chromosomes of the *P. gracilis* group, discussed in the previous section, is one such example. However, this type of data should be analyzed with caution, as heterochromatin includes satDNAs or other repetitive sequences characterized by accelerated evolutionary rates (Ugarković and Plohl 2002; Mehrotra and Goyal 2014; Garrido-Ramos 2017). In this context, the characterization of satDNA can be helpful.

The PcP190 satDNA has been mapped to the karyotypes of several Hyloidea species, contributing significantly to comparative cytogenetics within related clades (Vittorazzi 2014a, 2016; Gatto et al. 2018; Bueno et al. 2021). In our study, by analyzing specimens of *P. carrizorum* from Palmas-PR (Brazil), we found a chromosomal distribution of PcP190 satDNA clusters that differs from that previously reported for individuals from Misiones, Argentina (approximately 242 km from Palmas-PR). While the specimens from Misiones showed PcP190 satDNA clustered on chromosome 3, in specimens from Palmas this satDNA is clustered on chromosomes 1 and 3. Interpopulational variation in the number of conspicuous chromosomal satDNA clusters was previously reported by Vittorazzi et al. (2014a) for a genetic lineage of *P. cuvieri* usually referred to as lineage 2 (Lourenço et al. 2015; Nascimento et al. 2019). In specimens of lineage 2 from two localities (i.e., Uberlândia-MG and Três Lagoas-MS), PcP190 clusters were present in the (peri)centromeric region of all chromosomes except for chromosome 8, whereas specimens from Passo Fundo displayed strong signals of a PcP190 probe on chromosomes 1–5 and weak signals on some small chromosomes (Vittorazzi et al. 2014a). On the other hand, the number of PcP190 satDNA chromosomal clusters revealed by FISH in *P. ephippifer* is remarkably different from those observed in all genetic lineages recognized for *P. cuvieri* (Vittorazzi et al. 2014a).

In the case of *P. carrizorum* from Misiones and Palmas, in addition to the difference in the number of PcP190 chromosomal clusters, specimens from these localities also differ in the intrachromosomal position of the NOR and the distribution of heterochromatic blocks (as discussed above). Therefore, further taxonomic analyses are still needed to evaluate whether the cytogenetic differences reported here are due to intraspecific variation or reflect cryptic species. A PcP190 cluster on chromosome 1, similar to that observed in *P. carrizorum* from Palmas, was also found in *P. evangelistai* and *P. barrioi*, species closely related to *P. carrizorum*, whereas it was not found in *P. lisei* and *P. gracilis* (Ferro et al. 2022), which composed another clade in the *P. gracilis* group (see Fig. 5). Based on these findings, it is likely that the PcP190 cluster on chromosome 1 was lost in the *P. carrizorum* population from Misiones. However, whether the differential cytogenetic features observed in specimens from Misiones and Palmas play any role in the evolutionary divergence of these frogs remains to be elucidated.

### ﻿Chromosome mapping of the U2 snRNA gene in the *P. gracilis* group

Finally, the mapping of U2 snRNA gene also allowed a better characterization of the karyotypes in the *P. gracilis* group. Sites carrying U2 snRNA gene have already been mapped at the chromosomal level in other leptodactylid species, including species of *Physalaemus* (Souza et al. 2025) and *Leptodactylus* (Gazoni et al. 2021). In the *P. gracilis* group, U2 snRNA gene clusters are restricted to the terminal region of the short arm of chromosome 6 in the four analyzed species. Moreover, this condition (i.e., the presence of U2 snRNA gene cluster on chromosome 6) is also observed in other *Physalaemus* and *Leptodactylus* species (Gazoni et al. 2021; Souza et al. 2025), supporting the hypothesis proposed by Souza et al. (2025) of homology between chromosome 6 among these species. However, mapping this sequence in other genera of the family is still required to draw more robust conclusions regarding the potential of U2 snRNA gene cluster as a reliable marker for identifying the homology of chromosome 6 in Leptodactylidae.

## ﻿Conclusion

The presence of a constitutive pericentromeric heterochromatin band on the short arm of the NOR-bearing chromosomes may reinforce the hypothesis of homology between chromosome 10 of *P. barrioi* and chromosome 8 of the other species of the *P. gracilis* group. In contrast to the conserved location of a cluster of the U2 snRNA gene on chromosome 6, the NOR and PcP190 markers varied across the lineages within this group, even between two different populations of *P. carrizorum*. These findings highlight the need for further analysis of the diversity harbored by this species.

## ﻿Author contributions

PHPM: Conceptualization, Investigation, Methodology, Validation, Visualization, Writing - original draft, review and editing. LHBS: Conceptualization, Methodology, Writing - review and editing. JMF: Supervision, Writing - review and editing. LBL: Conceptualization, Funding acquisition, Methodology, Project administration, Resources, Supervision, Writing - review and editing. All authors approved the final version of the manuscript.

## Supplementary materials

Supplementary material 1 Alignment of PcP190 satDNA monomers Data type tif Explanation note PcP190 sequences are identified by their GenBank accession numbers. Colored sites indicate nucleotides that differ from the most common sequence observed in the majority of samples. Primers (P190F and P190R) are indicated by the dark arrows. The dark green region corresponds to the conserved region, while the light green corresponds to the hypervariable region of PcP190 satDNA. https://binary.pensoft.net/file/1460289This dataset is made available under the Open Database License (http://opendatacommons.org/licenses/odbl/1.0/). The Open Database License (ODbL) is a license agreement intended to allow users to freely share, modify, and use this Dataset while maintaining this same freedom for others, provided that the original source and author(s) are credited.Pedro Henrique Pacheco Mosquini, Lucas Henrique Bonfim Souza, Juan Martín Ferro, Luciana Bolsoni Lourenço
