# Supplementary figures and images for: ﻿Comparative cytogenetics of the Physalaemus gracilis group (Anura, Leptodactylidae) with characterization of the karyotype of Physalaemus evangelistai Bokermann, 1967
Source: Comp Cytogenet. 2025 Nov 7;19:171–88. doi: 10.3897/compcytogen.19.171637 (PMC12619069; doi:10.3897/compcytogen.19.171637)

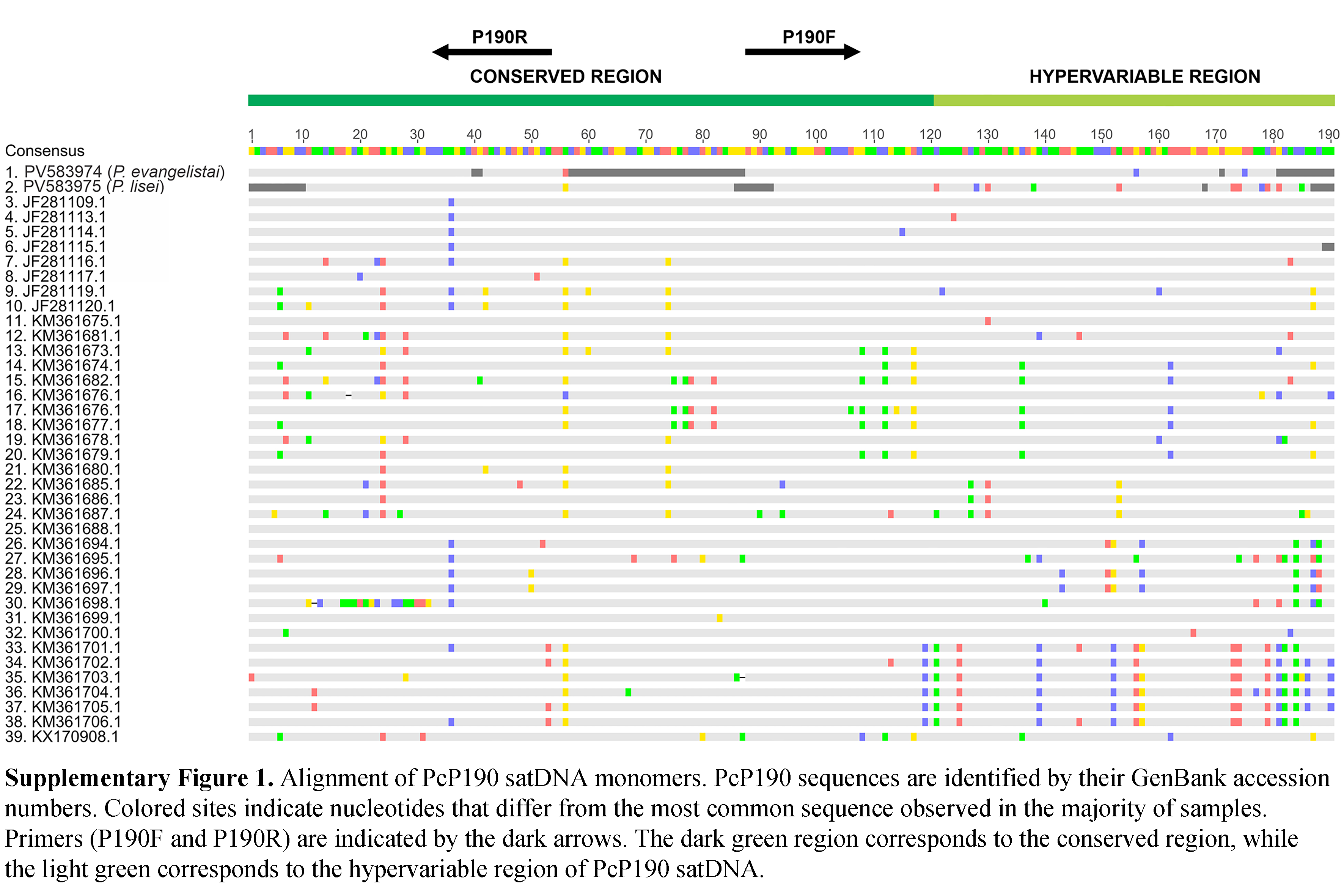

Supplement: Supplementary material 1 — Alignment of PcP190 satDNA monomers [file comparative_cytogenetics-19-171_article-171637__-s001.tif]
